# Evaluation of radiation maculopathy after treatment of choroidal melanoma with ruthenium-106 using optical coherence tomography angiography

**DOI:** 10.1186/s12886-021-02140-w

**Published:** 2021-11-02

**Authors:** Ali Torkashvand, Hamid Riazi-Esfahani, Fariba Ghassemi, Elias Khalili Pour, Babak Masoomian, Mohammad Zarei, Kaveh Fadakar, Mojtaba Arjmand, Fereshteh Tayebi, Leila Ekradi, Hamid Abrishami Moghaddam, Tahereh Mahmoudi, Reihaneh Daneshmand, Hooshang Faghihi

**Affiliations:** 1 Eye Research Center, Farabi Eye Hospital, Tehran University of Medical Sciences, Qazvin Square, Tehran, 1336616351 Iran; 2 Retina & Vitreous Service, Farabi Eye Hospital, Tehran University of Medical Sciences, Tehran, Iran; 3 Ocular Oncology Department, Farabi Eye Hospital, Tehran University of Medical Sciences, Tehran, Iran; 4 Machine Vision and Medical Image Processing (MVMIP) Lab, Faculty of Electrical Engineering, K.N. Toosi University of Technology, Tehran, Iran; 5 Department of Medical Physics and Biomedical Engineering, Tehran University of Medical Sciences and Research Center for Science and Technology in Medicine, Tehran, Iran; 6 Department of Biomedical Engineering, Amirkabir University of Technology, Tehran, Iran

**Keywords:** Deep capillary plexus, Foveal avascular zone (FAZ), Radiation maculopathy, Radiation retinopathy, Retina burnout, Ruthenium-106, Superficial capillary plexus

## Abstract

**Background:**

To assess the impact of brachytherapy on macular microvasculature utilizing optical coherence tomography angiography (OCTA) in treated choroidal melanoma.

**Methods:**

In this retrospective observational case series, we reviewed the recorded data of the patients with unilateral extramacular choroidal melanoma treated with ruthenium − 106 (^106^Ru) plaque radiotherapy with a follow-up period of more than 6 months. Automatically measured OCTA retinal parameters were analysed after image processing.

**Results:**

Thirty-one eyes of 31 patients with the mean age of 51.1 years were recruited. Six eyes had no radiation maculopathy (RM). From 25 eyes with RM, nine eyes (36%) revealed a burnout macular microvasculature with imperceptible vascular details. Twenty-one non-irradiated fellow eyes from the enrolled patients were considered as the control group. Foveal and optic disc radiation dose had the highest value to predict the burnout pattern (ROC, AUC: 0.763, 0.727). Superficial and deep foveal avascular zone (FAZ) were larger in irradiated eyes in comparison to non-irradiated fellow eyes (1629 μm^2^ vs. 428 μm^2^, *P* = 0.005; 1837 μm^2^ vs 268 μm^2^, *P* = 0.021; respectively). Foveal and parafoveal vascular area density (VAD) and vascular skeleton density (VSD) in both superficial and deep capillary plexus (SCP and DCP) were decreased in all irradiated eyes in comparison with non-irradiated fellow eyes (*P* < 0.001). Compared with non-irradiated fellow eyes, irradiated eyes without RM had significantly lower VAD and VSD at foveal and parafoveal DCP (all *P* < 0.02). However, these differences at SCP were not statistically significant.

**Conclusion:**

The OCTA is a valuable tool for evaluating RM. Initial subclinical microvascular insult after ^106^Ru brachytherapy is more likely to occur in DCP. The deep FAZ area was identified as a more critical biomarker of BCVA than superficial FAZ in these patients.

## Background

Ocular melanoma is the most common primary intraocular malignancy [1, 2]. Brachytherapy and teleradiotherapy have been used in the treatment of choroidal melanoma in the last decades. Both modes cause microvascular injuries in the retina and optic nerve, resulting in macular and optic disc edema, retinal ischemia, retinal hemorrhage, and neovascularization [3, 4].

Radiation retinopathy (RR), first described by Stallard [5], is characterized by irreversible endothelial cell damages leading to progressive occlusive vasculopathy [6]. It seems that small and deep vessels are more susceptible to this damage than large and superficial retinal vessels [7].

In 1300 patients with posterior uveal melanoma treated with ^125^I (Iodide-125) plaque brachytherapy, Gündüz et al. reported a rate of 5 and 43% of RR based on fundus photography (FP) and fluorescein angiography (FA), at 1 and 5 years, respectively [8]. Using optical coherence tomography (OCT) in 135 uveal melanoma patients treated with ^125^I brachytherapy, Horgan et al. demonstrated that the incidence of macular edema was 17, 40, and 61% at 6 months, and 1 and 2 years thereafter, correspondingly [9]. Another study showed that macular edema can be detected by OCT even 4 months after irradiation which is nearly 5 months earlier than ophthalmoscopic detection [10].

Optical coherence tomography angiography (OCTA) has recently emerged as a novel, fast, non-invasive, and reproducible imaging modality to evaluate the microvascular status in the macular region. It provides high-resolution quantitative data of both superficial and deep capillary plexus (SCP and DCP) within the macular region [11–13]. Changes in capillary density in the macular area after ^125^I brachytherapy have been investigated by OCTA in few studies [11–13]. Based on these reports, irradiated eyes had more sectors of non-perfusion areas and microaneurysms, as well as an enlarged foveal avascular area (FAZ) in both SCP and DCP. Even in eyes without apparent radiation maculopathy (RM), OCTA has shown significant reduction of capillary density in SCP and DCP [14].

Widely adopted in Europe, Ruthenium-106 (^106^Ru) is an alternative isotope to ^125^I for brachytherapy of uveal melanoma [15]. This β-emitting radioisotope has a threefold faster dose fall-off a greater lateral constriction than gamma-emitting ^125^I, with increasingly lower relative energies for every millimetres of target tissue thickness and normal surrounding tissues [15–17]. However, the consequences of ^106^Ru plaque brachytherapy on macular vasculature have not been comprehensively assessed by OCTA.

In this study, we evaluated the macular OCTA metrics following brachytherapy with ^106^Ru plaque for choroidal melanoma in comparison to the non-irradiated fellow eye, to explore the impact of ^106^Ru plaque on macular microvasculature.

## Methods

Our retrospective observational case series have been approved by Farabi Eye Hospital Institutional Review Board and Ethics Committee. The study adhered to the tenets of the Declaration of Helsinki. Informed consent was obtained from all participants.

### Participants

Between 1 February 2019 and 1 January 2020, consecutive patients treated with ^106^Ru brachytherapy for unilateral extramacular uveal melanoma were enrolled in the study. Surgical details of treatment have already been published elsewhere [18].

Bilateral same-day OCT and OCTA (Optovue Inc., Fremont, CA) were performed for every patient. For the patients with no evidence of RM in clinical examination and OCT/OCTA, fluorescein angiography (Heidelberg Engineering, Heidelberg, Germany) was performed to obtain more details.

RM was defined as the presence of macular edema (cystoid or non-cystoid), retinal telangiectasia, microaneurysm, cotton wool spots, exudation, hemorrhage, vascular occlusions, capillary nonperfusion area, and/or neovascularization. RM was confirmed or ruled out based on the findings of three expert investigators (FG, AT, and HRE) after reviewing the clinical exams and imaging modalities. Based on the presence or absence of RM, treated eyes were classified into two categories: eyes with RM and eyes without RM. The control group was selected from non-irradiated fellow eyes of enrolled patients.

The inclusion criteria were unilateral extramacular choroidal melanoma treated by ^106^Ru brachytherapy with a follow-up period of more than 6 months. The exclusion criteria were macular location of the tumor, presence of diabetic or hypertensive retinopathy, the history of other retinal vascular disorders (e.g., retinal vascular occlusion), glaucoma, uveitis, macular disorders (age related macular degeneration or choroidal neovascularization), retinal dystrophies and pan-retinal photocoagulation in either eye, previous vitreoretinal surgery, ocular trauma, eyes with visual acuity less than 20/200, refractive error > + 3 and < − 3 and significant media opacity precluding quality imaging. Eyes with low image quality (signal strength index (SSI) less than 50 according to built-in RTVue software quality assessment report) or different artifacts (including movement, shadow, decentration artifacts and, defocus) preventing accurate measurement of the vascular/skeleton density or FAZ area were excluded. The above exclusion criteria were also considered to select the non-irradiated fellow eyes as a control group.

Demographic data and radiation parameters including radiation dose to tumor apex and base, foveola, and optic disc (Gy) were collected from medical documents. The data additionally comprised pre-treatment tumor parameters obtained by indirect ophthalmoscopy including tumor location, distance to the fovea and disc, and tumor features detected by B-scan (largest tumor diameter and thickness). Subsequent consolidation therapies for tumor (transpupillary thermotherapy-TTT) and treatments directed for radiation side effects (intravitreal bevacizumab injection and sector laser photocoagulation) were also documented.

### Imaging acquisition protocol

All OCT and OCTA images were taken by Optovue RTVue XR AVANTI (Optovue Inc., Fremont, CA) device. For OCT scans, the device uses an 840-nm wavelength laser with a 3-mm scan width at the macular area and full width at half maximum of bandwidth of 45 nm to acquire 70,000 A-scans per second. For every location on the macula, the RTVue system captures two consecutive B-scans (M-B frames) each containing 304 A-scans (304 B-scan locations, each separated by 9.9 μm) [19]. Central foveal thickness (CFT) was documented.

The scanning algorithm for OCTA image acquisition starts with 2 B-scans taken before the next sampling location at each fixed spot and two orthogonal OCTA volume scans (one horizontal and one vertical) were taken to reduce fixation changes and motion artifacts. Split-spectrum amplitude-decorrelation angiography algorithm (SSADA) and projection artifact removal (PAR) algorithm is an integral module in Angio-Analytics software (version 2017.1.0.151) [19–21]. The segmentation of different layers of the retina was automatically performed. The boundaries of retinal slab for SCP were defined as 3 μm below ILM to 15 μm below the inner plexiform layer (IPL)-inner nuclear layer (INL) junction. The boundary of retinal slabs for DCP was defined as 15 μm to 70 μm below IPL-INL junction. All images were reviewed by two assessors (H.R.E and A.T) for image quality and segmentation errors. The segmentations were manually corrected or imaging was repeated, if necessary. If the assessors’ grading differed, a third opinion was sought (FG). The choroidal flow was automatically measured and documented.

The eye was classified as a ‘burnout’ case if the radiation damage was so severe that the vascular structures were not identifiable in OCTA.

### Vessel density calculation

Obtained data were evaluated in the case and the control eyes. There were no variations in SCP and DCP vascular density in both study groups. We noticed that the software wrongly interpreted the increased noise signals in the irradiated eyes due to poor vision and improper fixation as vascular signals. We decided to use image processing to get meaningful results in order to overcome this challenge. All images were exported to MATLAB software R2019a (Mathworks, Inc., Natick, MA) for further image processing and analysis. In the pre-processing stage, the original image (365 × 365 pixels with 1 pixel border around the image) was converted to the grayscale and then cropped to 364 × 364 pixels to remove the image borders in all images. After applying the homomorphic filter and normalization, an area within FAZ was manually selected in each image. Then the average of all pixel values in this area was computed to establish a threshold for being globally subtracted from the original image. Subsequently, the morphological top-hat and bottom-hat operations were applied. This was performed using a disc structural element with a radius of 4 pixels. Then, a bilateral filter was applied for edge preservation and noise reduction. In the next step, a Hessian vesselness filter proposed by Jerman et al. [22], was employed to improve the contrast of vessels.

In the second stage, an Otsu algorithm was applied for the detection of retinal vessels in SCP and DCP [23]. This algorithm uses a bi-modal histogram to find the optimum threshold in the image. Consequently, a binary image was constructed which included the location of vessels in SCP and DCP. To quantify the retinal vasculature, the skeleton of the image was required. Therefore, the skeletonization was implemented to iteratively thin the segmented vessels until a series of connected lines with a thickness of one pixel remained.

In the final stage, vessel area density (VAD) and vessel skeleton density (VSD) was calculated. VAD is calculated as a unitless ratio of the total image area occupied by the vasculature to the total image area in the binary vessel maps. VSD is calculated as the ratio of the length occupied by the blood vessels to the total area in the skeletonized vessel map [13].

**Fig. 1:**
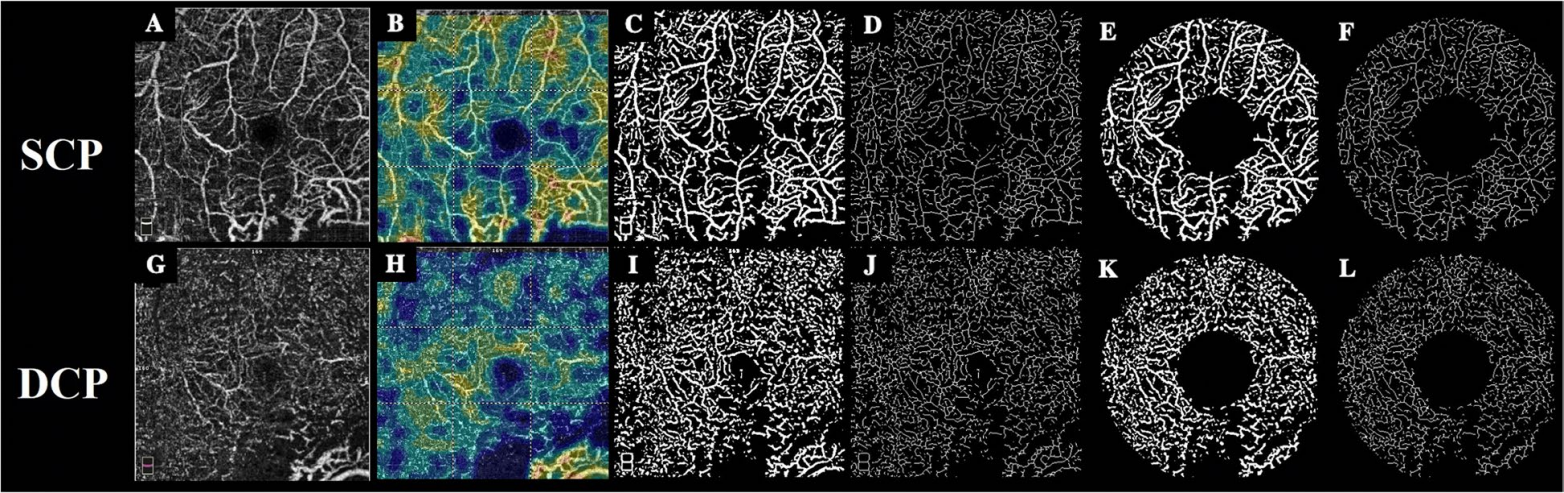
Original and processed images of superficial (upper row: **A** to **F**) and deep (lower row: **G** to **L**) capillary plexus of a 25-year-old patient, 14 months after plaque radiation. Figure **A** and **G** represents the original superficial capillary plexus (SCP) and deep capillary plexus (DCP) images, respectively. Figure **B** and **H** illustrates color-coded flow density map of the macula showing the SCP and DCP, respectively. (warmer colours indicate greater flow). Vessel density maps that are binarized images of SCP and DCP and produced after applying denoising and preprocessing algorithms on the original images have been shown in Figures **C** and **I**, respectively. Figures **D** and **J** show the skeletonization map-a series of connected lines with the thickness of one pixel that represents the route of vessels- in SCP and DCP, respectively. For calculation of the parafoveal vascular and skeleton density, two concentric circles were centered on the fovea with diameters of 1 mm and 3 mm. Figures **E** and **K** represent SCP and DCP parafoveal vascular density map and figures **F** and **L** show the skeletonization map in corresponding images

For calculation of foveal and parafoveal VAD and parafoveal VSD, two concentric circles were centered on the fovea with diameters of 1 mm and 3 mm. Then vessel density was calculated in the obtained ring (Fig. 1).

### FAZ area extraction

**Fig. 2:**
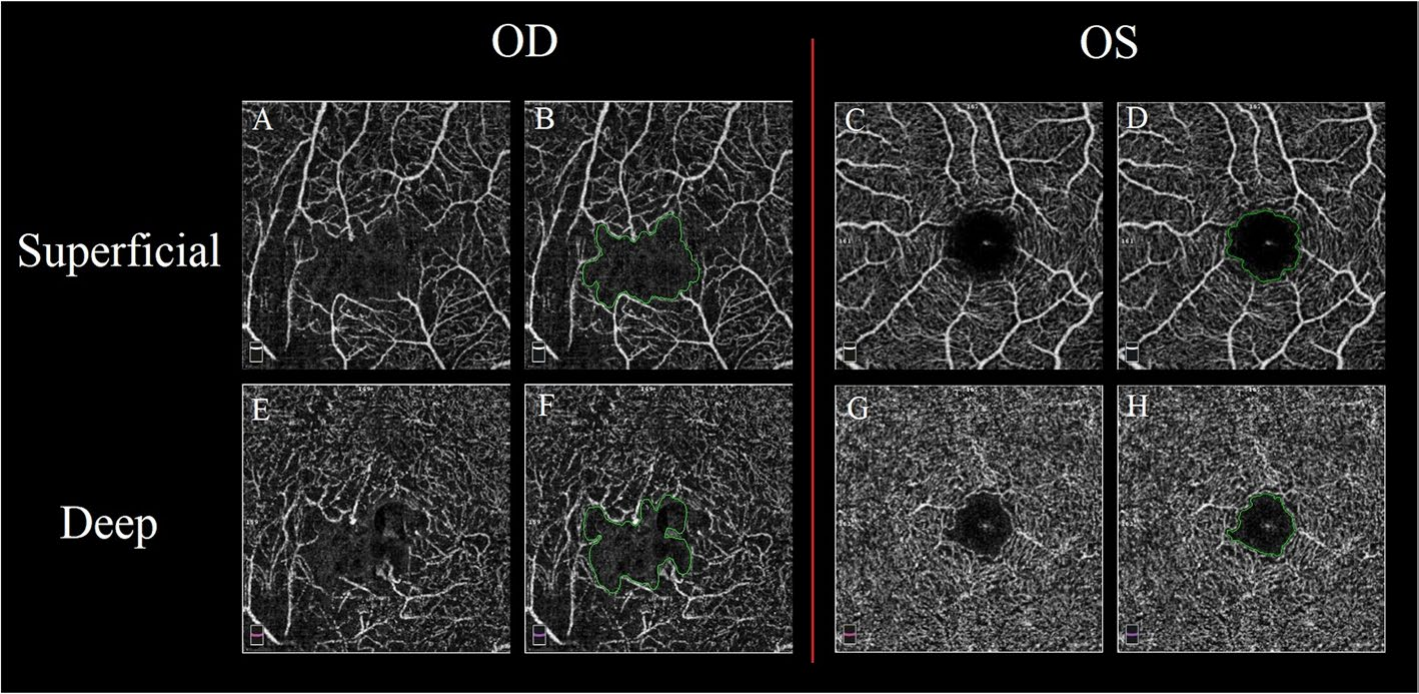
Foveal avascular zone extraction in superficial capillary plexus (SCP) and deep capillary plexus (DCP) images of right and left eye of a 52-year-old patient with choroidal melanoma who underwent plaque radiotherapy 30 months earlier. Figures **A** and **C** show original SCP images and figures **E** and **G** represent original DCP images of right and left eye, respectively. The green line in Figures **B** and **D** shows the foveal avascular zone (FAZ) area in SCP images and in figures **F** and **H** represent the FAZ area in DCP images, respectively

The FAZ area extraction algorithm was implemented in python using OpenCV and skimage libraries. The first step of FAZ area extraction was binarizing raw images with a variable threshold. The threshold was calculated as only the pixels representing the vessel’s margin that were identified by the algorithm and therefore the noise and motion artifacts were excluded. After the binarization of the image, some small white dots persisted in the center of the FAZ region that interfered with the automated determination of the FAZ region margins. These dots were eliminated from the image by morphological hole removal operations. The resulting images were then morphologically opened with a square or rectangular shaped structural elements to link the edges of the detected vessels in the image to form the FAZ area. Finally, the largest connected component that was nearest to the center of the image was selected as the FAZ and the area was calculated in mm^2^ based on image size (Fig. 2).

### Statistical analysis

All statistical analysis were performed by SPSS software (IBM Corp. Released in 2017. IBM SPSS Statistics for Windows, Version 25.0. Armonk, NY: IBM Corp.). Quantitative data was described as mean ± SD, and a normality test was performed for each variable. We used generalized estimation equation (GEE) to compare vascular indices between two eyes considering inter-eye correlation. Spearman rank correlation method was applied to evaluate the correlation of the variables. Receiver Operating Characteristic analysis (ROC) was performed to define the best variable that could predict the burnout condition in the eye. Logistic regression analysis was used to evaluate the effect of variables on development of RM in irradiated eyes. A *p*-value of less than 0.05 was considered statistically significant.

## Results

**Fig. 3:**
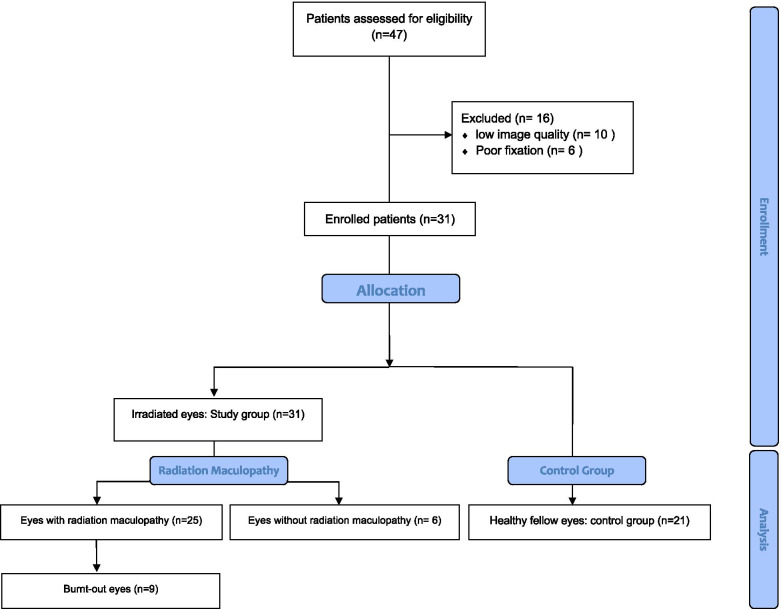
Consolidated Standards of Reporting Trial Diagram of Study Participant Disposition

From 47 patients who had been undergone ^106^Ru brachytherapy for extramacular choroidal melanoma, 16 eyes were excluded due to low-quality OCTA image or poor fixation. Therefore, 31 eyes of 31 patients were recruited in this study based on the inclusion and exclusion criteria (Fig. 3). The mean age of patients was 51.1 ± 14.6 years (range: 22–74 years) and 18 patients (56.8%) were female. The mean time period between brachytherapy and OCTA imaging was 32.8 ± 15.1 months (range: 6–62 months). The right eye was involved in 20 cases (62.5%).

All tumors were located in the extramacular area, with a mean largest tumor diameter of 14.0 ± 5.8 mm (range: 8.50–21) and a mean thickness of 6.7 ± 2.2 mm (range: 2.84-10 mm). The mean distance between the tumor and the fovea and optic disc was 3.5 ± 3.3 mm (range 2-12 mm) and 3.8 ± 3.4 mm (range 0–12 mm), correspondingly. The mean radiation dose to the fovea, optic disc, apex of the tumor, and sclera was 45.7 ± 97.5, 32.9 ± 53.6, 84.2 ± 9.2, and 525.2 ± 298.4 Gy, respectively. The median of BCVA in treated eyes was 0.71 LogMAR (interquartile 25–75: 0.30–1.00 LogMAR) at the time of image acquisition. Adjuvant treatments like TTT, sector retinal laser photocoagulation, and intravitreal bevacizumab were performed for 7(22.3%), 15(48.3%), and 19(61.2%) of the cases, respectively. Table 1 provides the baseline characteristics of the participants and treatment features.

**Table 1:** Baseline features of 31 participants and characteristics of the tumor and treatment with^106^Ru brachytherapy

**Features**	
**Mean Age (median, range-Y)**	51 (55, 22–74)
**Sex (F)**	18 (56.8%)
**Mean BCVA (median, range-LogMAR)**	0.71 (0.52, 0.00–3.00)
**Involved eye (OD)**	20 (62.5%)
**Mean distance to fovea (median, range-mm)**	3.53 (3.00, 2.00–12.00)
**Mean distance to optic disc (median, range-mm)**	3.81 (3.00, 0.00–12.00)
**Mean largest tumor diameter (median, range-mm)**	14.08 (14.00,8.50–21.00)
**Mean thickness (median, range-mm)**	6.75 (6.70, 2.84–10)
**Mean foveal dose (median, range-Gy)**	45.76 (11.00, 0.00–501)
**Mean optic disc dose (median, range-Gy)**	32.91 (13.50, 0.00–266)
**Mean apex dose (median, range-Gy)**	84.29 (85, 40–100)

Among the 31 eyes, six eyes (19.4%) had no signs of RM based on the funduscopy, FA, and OCT results and were classified as irradiated eyes without RM, while the remaining 25(80.6%) patients had evidence of maculopathy based on these tests. The mean interval between brachytherapy and OCTA imaging in eyes with and without RM was 34.3 ± 12.1 months and 15.4 ± 14.9 months, respectively.

### Burnout eyes

From 25 eyes with documented RM, nine eyes (36%) had serious attenuation of retinal vasculature and remarkable macular ischemia (more than 9mm^2^) with undetectable capillary plexus details in OCTA (burnout macula). Among the tumor characteristics and radiation parameters, the foveal and the optic disc radiation dose had the highest sensitivity and specificity to predict the burnout macula (ROC, AUC: 0.763 and 0.727, correspondingly).

### Irradiated vs non-irradiated eyes

Table 2 demonstrates the comparison of OCT and OCTA metrics between irradiated (16 eyes with RM but acceptable macular vascular structure and 6 eyes without RM) and non-irradiated fellow eyes (21 eyes). The central foveal thickness (CFT) was more in treated eyes in comparison with the non-irradiated fellow eyes, even though the difference was not significant (291.81 μm vs. 248.46 μm, *P* = 0.133). Superficial FAZ area was increased in treated eyes (1629 ± 206.3 μm^2^ vs 428 ± 778 μm^2^, *P* = 0.005). Deep FAZ area was also increased in treated eyes in comparison with non-irradiated fellow eyes. (1837 ± 225.2 μm^2^ vs 268 ± 120 μm^2^, *P* = 0.021). The foveal superficial VAD was lower in treated eyes (22.2 ± 8.3 vs 29.4 ± 3.8, *P* < 0.001). Comparatively, the parafoveal superficial VAD also showed a decrease in the treated eyes (23.54 ± 9.29 vs 32.13 ± 4.11, *P* < 0.001). Similarly, at DCP, the foveal VAD (23.2 ± 9.3 vs 34.6 ± 3.5, *P* < 0.001) and parafoveal VAD (24.2 ± 9.9 vs 36.5 ± 3.5, *P* < 0.001) were lower in irradiated eyes. The VSD was also decreased in both SCP and DCP in the fovea and parafovea area of the irradiated eyes (*P* < 0.001 for all). (Table 2) Choriocapillaris flow area was significantly lower in treated eyes (1.9 ± 0.2 vs 2.1 ± 0.10, *P* < 0.001).

**Table 2:** Comparison of OCT and OCTA parameters in eyes with or without 106Ru brachytherapy for choroidal melanoma

		**Treatment status**		**Difference (CI95%)**	***P*-value†**
		Yes (*n* = 22)	No (*n* = 21)		
**Foveal thickness μm**		291.81 ± 144.13	248.46 ± 35.08	−44.16 (− 101–13.40)	0.133
**FAZ μm**^**2**^	Superficial	1629 ± 2063	428 ± 778	− 1200 (− 2036–− 363)	**0.005**
	Deep	1837 ± 2252	268 ± 120	− 1569 (− 2504–− 634)	**0.001**
**Superficial vascular density %**	Fovea	22.22 ± 8.33	29.43 ± 3.81	7.24 (3.64–10.84)	**< 0.001**
	Parafovea	23.54 ± 9.29	32.13 ± 4.11	8.58 (4.38–12.77	**< 0.001**
**Deep vascular density %**	Fovea	23.25 ± 9.38	34.67 ± 3.55	11.38 (7.15–15.61)	**< 0.001**
	Parafovea	24.29 ± 9.91	36.58 ± 3.54	12.19 (7.57–16.81)	**< 0.001**
**Choriocapillaris flow area mm**^**2**^		1.92 ± 0.26	2.12 ± 0.10	0.20 (0.09–0.30)	**< 0.001**
**Superficial skeleton density %**	Fovea	8.36 ± 3.43	11.46 ± 1.54	3.13 (1.73–4.53)	**< 0.001**
	Parafovea	8.80 ± 3.84	12.47 ± 1.65	3.69 (2.06–5.32)	**< 0.001**
**Deep skeleton density %**	Fovea	10.03 ± 4.11	15.18 ± 1.55	5.14 (3.30–6.98)	**< 0.001**
	Parafovea	10.29 ± 4.37	15.90 ± 1.53	5.56 (3.55–7.57)	**< 0.001**

*FAZ* Foveal avascular zone

†Based on GEE analysis

Among the baseline tumor features, the best-corrected visual acuity (BCVA-LogMAR) was correlated to foveal dose (*r* = 0.386, *p* = 0.032) and deep FAZ area (*r* = 0.450, *p* = 0.036). Optic disc dose was correlated with superficial and deep FAZ (*r* = 0.447, *p* = 0.048; *r* = 0.599, *P* = 0.005; respectively) and showed an inverse correlation with superficial foveal and parafoveal vascular density (*r* = − 0.482, *p* = 0.023; *r* = − 0.485, *P* = 0.022; respectively).

### Eyes with vs without radiation maculopathy

Table 3 illustrates associations of various factors with RM in irradiated eyes. The foveal and parafoveal VAD and VSD of SCP were lower in eyes with RM; however, this difference was not statistically significant. The same pattern was observed for DCP foveal and parafoveal vascular indexes. The time interval between plaque implantation and image acquisition had a direct modest correlation with RM (34.3 ± 12.1 months vs 15.4 ± 14.9 months, OR: 1.124, 95%CI: 1.021–1.239; *P* = 0.017). An inverse marginal association was observed between the tumor to fovea distance and the presence of RM (5.67 ± 3.88 vs 3.00 ± 3.02, OR: 0.795, 95%CI: 0.608–1.040; *P* = 0.094). In multivariate regression analysis after adjusting the effect of age and sex, the time interval between plaque implantation and image acquisition was still significantly associated with RM (OR: 1.118, 95%CI: 1.015–1.231; *P* = 0.024).

**Table 3:** Association of various parameters with the presence of radiation retinopathy in irradiated eyes

		**Radiation Retinopathy**		**Odds Ratio (Confidence Interval 95%)**	***P*-value†**
		Yes (*n* = 16)	No (*n* = 6)		
**Age**		51.75 ± 14.11	49.00 ± 16.68	1.013 (0.956–1.074)	0.654
**Foveal thickness μm**		319 ± 166	237 ± 64	1.004 (0.996–1.012)	0.319
**FAZ mm**^**2**^	Superficial	1775 ± 2127	311 ± 262	1.005 (0.993–1.016)	0.411
	Deep	2023 ± 2303	168 ± 74	1.019 (0.983–1.055)	0.306
**Superficial vascular area density %**	Fovea	20.75 ± 8.72	26.14 ± 6.15	0.913 (0.798–1.044)	0.183
	Parafovea	21.98 ± 9.85	27.70 ± 6.54	0.925 (0.820–1.043)	0.204
**Deep vascular area density %**	Fovea	22.23 ± 9.94	25.99 ± 7.80	0.955 (0.859–1.062)	0.397
	Parafovea	23.20 ± 10.43	27.19 ± 8.50	0.957 (0.865–1.059)	0.396
**Superficial vascular skeleton density %**	Fovea	7.76 ± 3.65	9.95 ± 2.29	0.806 (0.585–1.111)	0.189
	Parafovea	8.16 ± 4.12	10.49 ± 2.50	0.834 (0.627–1.108)	0.210
**Deep vascular skeleton density %**	Fovea	9.51 ± 4.37	11.42 ± 3.24	0.886 (0.694–1.131)	0.330
	Parafovea	9.74 ± 4.63	11.77 ± 3.48	0.891 (0.707–1.123)	0.327
**Choriocapillary flow area density mm**^**2**^		1.88 ± 0.28	2.07 ± 0.14	0.019 (0.000–4.470)	0.156
**Follow-up time (m)**		34.33 ± 12.17	15.43 ± 14.98	1.124 (1.021–1.239)	**0.017**
**Foveal dose (Gy)**		20.50 ± 29.60	55.42 ± 110.53	1.009 (0.986–1.032)	0.445
**Apex dose (Gy)**		83.42 ± 9.51	87.29 ± 7.78	0.889 (0.720–1.097)	0.273

*FAZ* Foveal avascular zone

†Based on logistic regression analysis

In the subgroup of eyes that did not have any signs of RM at the time of acquisition of OCTA, the disparity between mean CFT in irradiated and corresponding non-irradiated fellow eyes was not statistically significant (*P* = 0.707). The FAZ area was larger in the irradiated eyes compared to the non-irradiated fellow eyes, but these differences for superficial (*P* = 0.292) and deep FAZ (*P* = 0.689) were not significant.

In this subgroup, VAD and VSD at SCP in both foveal and parafoveal areas of the irradiated eyes were lower, but this differences for superficial VAD (*P* = 0.282 and *P* = 0.200, respectively) and superficial VSD of foveal and parafoveal area (*P* = 0.091 and *P* = 0.064, respectively) were not significant. In contrast, in the DCP region, the foveal (*P* = 0.014) and parafoveal VAD (*P* = 0.016) were significantly decreased in irradiated eyes compared to non-irradiated fellow eyes. A significant difference in both foveal and parafoveal VSD was also detected (*P* = 0.010 for both) (Table 4).

**Table 4:** The OCTA features of irradiated eyes without radiation retinopathy in comparison with the non-irradiated fellow eyes in the patients with choroidal melanoma

		**Treatment status**		**Difference (CI95%)**	***P*-value†**
		Yes (*n* = 6)	No (*n* = 6)		
**Retinal foveal thickness μm**		237 ± 64	231 ± 61	−5.50 (− 34.19–23.19)	0.707
**FAZ μm**^**2**^	Superficial	612 ± 626	537 ± 447	− 75.20 (− 215–64.81)	0.292
	Deep	339 ± 320	346 ± 333	7.22 (−28.17–42.61)	0.689
**Superficial vascular density %**	Fovea	26.14 ± 6.15	28.70 ± 4.26	2.55 (− 2.09–7.20)	0.282
	Parafovea	27.70 ± 6.54	31.04 ± 4.71	2.60 (− 1.77–8.45)	0.200
**Deep vascular density %**	Fovea	25.99 ± 7.80	34.28 ± 3.22	8.28 (1.65–14.91)	**0.014**
	Parafovea	27.19 ± 8.50	36.19 ± 2.90	9.00 (1.68–16.32)	**0.016**
**Superficial skeleton density %**	Fovea	9.95 ± 2.29	11.31 ± 1.71	1.35 (− 0.21–2.93)	0.091
	Parafovea	10.49 ± 2.50	12.18 ± 1.88	1.68 (− 0.09–3.47)	0.064
**Deep skeleton density %**	Fovea	11.42 ± 3.24	15.10 ± 1.41	3.68 (0.89–6.46)	**0.010**
	Parafovea	11.77 ± 3.48	15.76 ± 1.39	3.98 (0.93–7.02)	**0.010**

*FAZ* Foveal avascular zone

†Based on GEE analysis

## Discussion

The current study revealed that DCP is likely to develop the earliest subclinical radiation-induced microvascular insult following ^106^Ru plaque brachytherapy. The deep FAZ area was identified as a more critical determinant of BCVA than superficial FAZ in these patients. Among the tumor characteristics and radiation parameters, the foveal dose and the optic disc dose had the highest sensitivity and specificity to predict the burnout pattern of the retinal microvasculature. Choriocapillaris flow area was significantly decreased in the treated eyes.

RR may lead to visual morbidity and blindness following choroidal melanoma brachytherapy, in fact in cases of maculopathy [15–17]. The Collaborative Ocular Melanoma Study Group (COMS-report No.16) recorded 6 lines of vision loss in 49% of patients who were treated with ^125^I brachytherapy after 3 years [20]. In 43% of patients with an initial vision of 20/200 or better at the time of diagnosis, vision declined gradually to 20/200 or worse by 3 years [24].

According to previous studies, the risk of RR following brachytherapy is directly linked to the overall dose of administered radiation [25]. In the treatment of choroidal melanoma, the dose of radiation to the apex of the tumor is between 62 and 104 Gy based on various studies [26]. Other factors, such as tumor height and diameter as well as the position of the tumor, are correlated with the risk of retinopathy [25]. Our results showed that the time interval between plaque implantation and image acquisition is the only independent factor predicting RM based on fundoscopy, FA, and/or OCT findings (Table 3).

To date, RM diagnosis has been primarily based on biomicroscopic and angiographic data or OCT findings of macular edema [10]. Few studies investigated the role of OCTA in the early detection of RM. Previous studies in patients treated with ^125^I plaques have shown that OCTA is probably the most effective existing imaging for detecting early signs of RM [11–14]. OCTA offers a 3-dimensional volumetric scan that displays the segmented distribution of the blood in the macular area—something that is not possible with conventional FA. All of our patients were treated with ^106^Ru plaque brachytherapy. The study showed that most of the examined OCTA-derived metrics, including the vascular density and FAZ in the SCP and DCP, had been altered in irradiated eyes compared to non-irradiated eyes.

According to this report, it appears that the evaluation of the crude data directly obtained from the OCTA instrument is not appropriate for the assessment of vascular density of capillary plexus in the macular area due to the high rate of noises obscuring the information. As reported in previous studies, imaging artifacts and noises may induce some signals and may influence the vascular density and FAZ measurement [12, 14]. Despite higher speed of current OCTA machines, these artifacts are usually present in irradiated eyes because of low vision and resulted fixation deficit and apparent structural changes. It is noteworthy that OCTA artifacts are more common in eyes treated by brachytherapy than untreated eyes [14]. Although improvements in density measurement could be attained with repeated imaging and fixation aids in some cases, image processing, noise reduction, and binarization are usually needed to alleviate this problem. In this study, the images were processed with sufficient filters to generate high-quality valid data for analysis.

By measuring the skeleton mass of vessels and minimizing the weight of large vessels, the analysis was made more specified for small vessels that are possibly most commonly affected by radiation [13]. The endpoints assessed after noise reduction and image processing indicated macular capillary plexus disorganization and obliteration after ^106^Ru brachytherapy. Since the manual segmentation and analysis of the images could be a possible source of variability, we designed automated methods to measure biomarkers like VAD, VSD and FAZ area as the strength of this study.

Although few studies have reported OCTA results after 125I brachytherapy, no comparable comprehensive study after 106Ru brachytherapy is available [3, 4, 11, 12, 14, 27, 28]. Veverka et al. [29] showed gradual alterations of the macular microvasculature on OCTA following melanoma treatment with ^125^I plaque. Shields et al. [12] reported enlargement of the FAZ region and decreased capillary density in both SCP and DCP after ^125^I brachytherapy. In another study [14], the patients with choroidal melanoma treated with ^125^I plaque and normal macular ophthalmoscopy and OCT showed a statistically significant decrease in density of both SCP and DCP. Most of these studies signified the role of OCTA in the early detection of changes even in eyes without RM [12, 14, 27, 28]. According to the present research, DCP vascular density decrease (VAD and VSD) in foveal and parafoveal areas is the first biomarker for RM occurring prior to clinical and OCT and FA signs of RM. Consistently, Matet et al. [30] showed that after proton beam therapy, the DCP of irradiated eyes was altered more severely than the SCP. Using volume-rendering display, Spaide showed that in RM, macular edema is associated with DCP non-perfusion [31]. In our current study, skeleton density assessment confirmed more pronounced DCP changes in comparison to SCP in irradiated eyes. Skeleton density is a more accurate assessment of retinal vascular alterations because the skeletonized image normalizes the diameter of larger vessels with that of capillaries, eliminating the effect of vessel size on retinal perfusion measures [32].

We assume that in the earlier stage of RM, the measured loss of capillary density could be secondary to a decrease in flow velocity below the predetermined decorrelation threshold of the SSADA algorithm and not a true structural loss of the vessel. Histopathological and ultrastructural studies of the human retina following radiation are extremely scarce and mainly report changes with varying degrees of retinal ischemia and atrophy in the larger retinal and choroidal vessels. Histologic studies have also confirmed the early and preferentially loss of vascular endothelial cells leading to occlusion of capillaries in which pericytes still survived [33]. Endothelial cell loss is due to impaired cell division and free radical production [3, 4, 25]. More severely affected retina revealed acellular capillaries with the residual basement membrane tubes being typically fused, shrunken, or collapsed [6]. It can be concluded that slower than normal cellular (red and white blood cells) flow in the basement membrane walled tubes could be detected as lower vascular density in both SCP and DCP in the irradiated parts of the retina.

Radio-sensitivity of the DCP is higher than that of the SCP. The smaller capillaries in DCP are more radiosensitive than larger capillaries in SCP [34]. The lower perfusion pressure in these capillaries as terminal vessels make them more vulnerable to occlusion after endothelial cell damage or loss. Moreover, studies suggest that blood flows from SCP through serial connections of vertically descending anastomoses and vortex-like channels to the DCP [35, 36]. Capillaries of DCP are likely to be terminal vessels and tend to be more sensitive than SCP to ischemic stress, similar to terminal capillaries in other organs, as kidney [36, 37]. Even slight changes in retinal circulation may also primarily affect DCP.

Some studies, evaluating other retinal vascular disorders such as retinal vein occlusion and diabetic retinopathy, have shown that reduced perfusion is more common in DCP than SCP [34, 35]. On the other hand, the DCP non-perfusion has recently been identified as a more critical determinant of BCVA than SCP nonperfusion in patients with retinal vascular disorders [38, 39]. We also revealed that the deep FAZ was significantly correlated with BCVA. Finally, DCP flow derives exclusively from SCP, therefore it may receive a larger amount of downstream inflammatory or free radicals from the upstream part of SCP after irradiation [30].

It seems that ^106^Ru is less destructive to the fovea and optic disc compared to ^125^I due to a higher dose gradient through the tissue and shorter penetration and lateral distribution [16, 18, 40]. No study has yet compared vascular changes found in OCTA characteristics of retina and choroid following brachytherapy with ^125^I and ^106^Ru plaques. Despite a higher dose of radiation to the tumor apex (84.29 Gy vs. 71.5 Gy) in our study compared to the report by Shields et al. [14], the mean dose of radiation to fovea and optic disc was lower (45.76 Gy vs 50.6 Gy and 32.5 Gy vs 40.3 Gy, respectively). The quantitative comparison of our findings with the detailed features reported by Shields et al. [12] showed similar results.

From the eyes with documented RM, nine eyes (36%) had severe damages with very extensive macular ischemia in which capillary plexus detail was not detectable in OCTA (burnout). In ROC analysis, the foveal dose and optic disc dose were better parameters for predicting the burnout pattern. However, due to the insufficient number of cases, the macular tolerance threshold could not be calculated.

The optic disc dose was positively correlated with superficial and deep FAZ area and had an inverse correlation with foveal and parafoveal SCP vascular density. As a result, foveal and disc radiation doses appear to be the primary predictors of both macular microvascular damage and visual function (BCVA).

The limitations of our study are mostly related to the retrospective design and the small number of cases, and imaging acquisition. While extensive efforts have been made to remove the artifacts of OCTA images, the development of new algorithms has not been entirely successful in this period for patients with RM. The study removed low-quality images, a source of selection bias. This may lead to underestimation or oversimplification of the exact impact of radiation on vascular indices. Finally, cystic retinal alterations caused by RM may cause erroneous vascular and skeletal density assessments. Although we attempted to eliminate eyes with burnt-out macular changes, reliable and exact assessment of vascular metrics in individuals with intraretinal cystic abnormalities such as diabetic retinopathy and RM remains controversial [41].

Although we chose the patients who had extra-macular tumors, but adjuvant treatments potential impact was overlooked. According to recent studies, retinal capillary density and FAZ area remain statistically unchanged after intravitreal injection of an anti-VEGF agent or PRP in patients with diabetic retinopathy [42, 43]. In addition, we also have not assessed the status of the retinal vasculature at baseline and the longitudinal changes after treatment.

In this study, using suitable image processing software added more value to the analysis by reducing abnormal noise and more precise segmentation as a strength.

## Conclusions

In conclusion, initial subclinical microvascular insult after ^106^Ru plaque brachytherapy is more likely to occur in DCP. The deep FAZ area was identified as a more critical biomarker of BCVA than superficial FAZ in these patients. The foveal dose and the optic disc dose had the highest sensitivity and specificity among the tumor characteristics and radiation parameters to predict retinal microvascular burnout. Potential artifacts are more common in irradiated eyes with worse visual function, hence image processing seems to be necessary in these cases prior to image analysis. Future prospective chronologic studies focused on serial OCTA imaging are required to better understand the pathophysiology of RM.

## Data Availability

The datasets generated and/or analysed during the current study are not publicly available due to limitations of ethical approval involving the patient data and anonymity but are available from the corresponding author on reasonable request.

## Abbreviations

OCTA: Optical coherence tomography angiography
OCT: Optical coherence tomography
Ru-106: Rheuthenium-106
^125^I: Iodine 125
FAZ: Foveal avascular zone
VAD: Vascular area density
VSD: Vascular skeleton density
SCP: Superficial capillary plexuses
DCP: Deep capillary plexuses
RM: Radiation maculopathy
RR: Radiation retinopathy
PBR: Proton beam radiotherapy
CFT: Central foveal thickness
BCVA: Best corrected visual acuity
IPL: Inner plexiform layer
INL: Inner nuclear layer
TTT: Transpupillary thermotherapy
